# GM-CSF improves the immune response to the diphtheria-component in a multivalent vaccine

**DOI:** 10.1016/j.vaccine.2018.06.033

**Published:** 2018-06-28

**Authors:** Marco Grasse, Andreas Meryk, Carina Miggitsch, Beatrix Grubeck-Loebenstein

**Affiliations:** Institute for Biomedical Aging Research, Universitat Innsbruck, Innsbruck, Austria

**Keywords:** Diphtheria, Tetanus, Vaccination, GM-CSF, Antibody

## Abstract

Multivalent tetanus and diphtheria toxoid containing vaccines belong to the most frequently applied vaccines. However, there is an imbalance in the degree of protection against the two antigens with insufficient long-term protection against diphtheria, particularly in the elderly population. We have previously reported a positive correlation between granulocyte macrophage-colony stimulating factor (GM-CSF) and the production of diphtheria-specific antibodies. Therefore, in the present study we analyzed the effects of *in vivo* applied recombinant GM-CSF on immunization with multivalent tetanus/diphtheria vaccine in mice of different age. In vivo application of GM-CSF lead to enhanced production of diphtheria-specific antibodies as well as more diphtheria-specific CD4^+^ T cells following vaccination with multivalent tetanus/diphtheria vaccine. In contrast, the humoral and cellular immune response to the tetanus component was unaltered. Furthermore, application of GM-CSF resulted in more splenic CD11b^+^ dendritic cells (DCs) with a higher MHC-II expression. GM-CSF also induced a stronger recruitment of CD11b^+^ DCs to the injected muscle. Most remarkably, GM-CSF was able to boost the diphtheria-specific immune response to the multivalent vaccine in aged mice. This study demonstrates that local administration of GM-CSF is able to improve immune responsiveness to the diphtheria component of multivalent tetanus/ diphtheria vaccine in young and old mice. This information could be useful for the future design of vaccines for the elderly.

## 1 Introduction

Immunization is a powerful weapon for the prevention of infectious diseases, such as smallpox, tetanus or diphtheria. Diphtheria vaccines belong to the most frequently applied vaccines worldwide, with a long history dating back to the 1920s, when Glenny and Hopkins inactivated the diphtheria toxin and showed a high immunity index of the toxoid [1]. In the early 1930s the diphtheria toxoid was found to be more immunogenic when adsorbed to aluminum salts as carrier [2]. To date, no drastic changes have been made to the vaccine. The toxoid is formulated with aluminum-hydroxide as an adjuvant in multivalent vaccines combined with other antigens, such as pertussis, polio and the most prevalent, tetanus toxoid.

Our group has a long-standing history studying tetanus and diphtheria vaccinations in persons of different ages and we have repeatedly shown good protection against tetanus, while protection against diphtheria was insufficient [3–5]. Studies by other groups have also demonstrated insufficient protection against diphtheria in adults [6–8]. Therefore we were curious how one could improve the immune responsiveness to the diphtheria component of the vaccine, which is of particular importance for elderly persons who are unprotected against diphtheria, more frequently than young [5,9]. Increasing the diphtheria dosage is not a viable option due to reported side effects of booster vaccines containing high diphtheria toxoid concentrations [10,11]. In a recent study we found a positive correlation between diphtheria-specific granulocyte macrophage-colony stimulating factor (GM-CSF) production by CD4^+^ T cells, and peripheral diphtheria-specific antibodies in adults [5]. GM-CSF is a cytokine mainly produced by activated leukocytes and recognized, via the GM-CSF receptor, by granulocytes, monocytes, macrophages, as well as DCs and their precursors [12,13]. GM-CSF is known to stimulate chemotaxis, proliferation and differentiation, and is also generally recognized as a pro-inflammatory cytokine [14,12,15]. There are numerous studies that successfully tested GM-CSF as an adjuvant for vaccines, thereby improving immune responses to H5N1 influenza virus, a crude leishmania antigen vaccine, hepatitis B virus, human immunodeficiency virus (HIV), *Mycobacterium tuberculosis*, and many more [16–22].

The goal of the present study was to investigate the *in vivo* effect of GM-CSF on immune responses to diphtheria and tetanus immunization in young and aged mice using multivalent vaccines containing diphtheria toxoid, tetanus toxoid, acellular pertussis, and inactivated polio virus, to model the human immunization schedule.

## 2 Materials and methods

### 2.1 Animals

Eight week and 17 month old C57BL/6JRj male mice were purchased from JANVIER LABS (Le Genest-Saint-Isle, France). Mice were provided standard food and water *ad libitum*, and kept under specific pathogen free conditions. All animal protocols were approved by the Federal Ministry of Science, Research and Economy of Austria, and carried out in accordance with the Austrian law for animal protection and the institutional guidelines at the University of Innsbruck.

### 2.2 Immunization

**Fig. 1:**
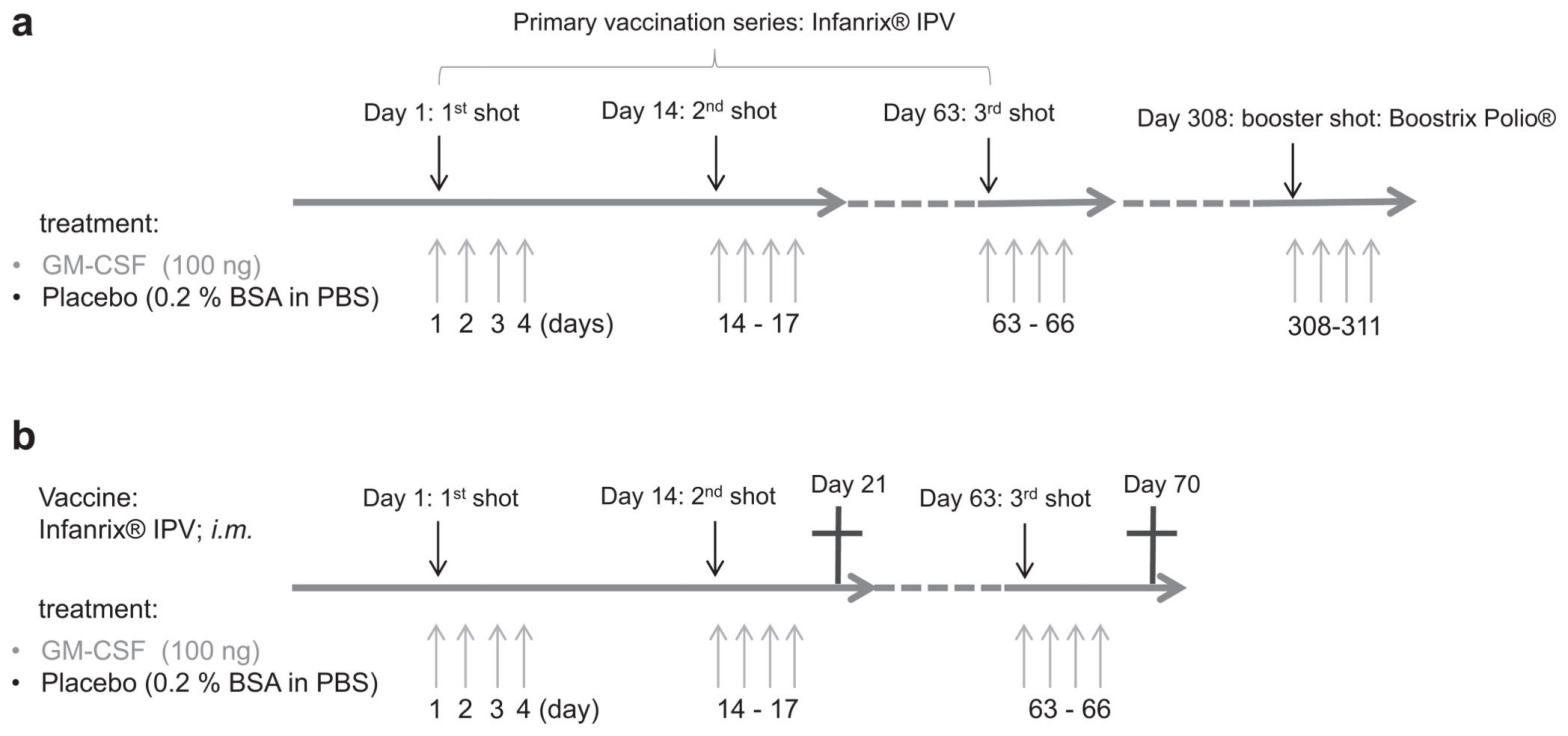
Immunization schemes with diphtheria- and tetanus-toxoid containing vaccines. (a) Mice received a primary immunization with three shots of Infanrix® IPV within 9 weeks, and one booster shot with Boostrix Polio® 9 months thereafter. The vaccines were injected *i.m*. into the *Musculus bicepsfemoris* of the right leg. At each vaccine-shot, and the consecutive three days, 100 ng of GM-CSF (diluted in PBS with 0.2% BSA) or a placebo (PBS with 0.2% BSA) was injected into the same site where the vaccine was applied. (b) Some mice received two and some received three shots of Infanrix® IPV (i.m. right leg) within 9 weeks, and at each vaccine-shot, and the consecutive three days, 100 ng of GM-CSF (diluted in PBS with 0.2% BSA) or a placebo (PBS with 0.2% BSA) was injected into the site where the vaccine was applied. Splenocytes were isolated 7 days after the last vaccine shot.

Mice were vaccinated with either 30 μL of diluted Infanrix® IPV containing not less than 30 IU diphtheria toxoid, and not less than 40 IU tetanus toxoid, three purified antigens of *Bordella pertussis* (25 μg pertussis toxoid (PT), 25 μg pertussis filamentous haemag-glutinin (FHA), and 8 μg pertactin), and three types of inactivated polio viruses (40 D-antigen units type 1: Mahoney strain; 8 D-antigen units type 2: MEF-1 strain; 32 D-antigen units type 3: Saukett strain; GlaxoSmithKline, London, United Kingdom) per single human dose (0.5 mL), or 30 μL of diluted Boostrix Polio® containing per single human dose (0.5 mL) not less than 2 IU diphtheria toxoid, and not less than 20 IU tetanus toxoid, three purified antigens of *Bordella pertussis* (8 μg PT, 8 μg FHA and 2.5 μg pertactin) and the same three types of inactivated polio viruses as included in Infanrix® IPV at the same dosages (GlaxoSmithKline) *i.m*. into the *Musculus biceps femoris* of the right or left leg. For all vaccinations, Infanrix® IPV from the same batch was used. The same was true for Boostrix Polio®. Both vaccines were diluted 1:2.4 with phosphate buffered saline (PBS; Merck KGaA, Darmstadt, HE, Germany). GM-CSF-treated mice received 100 ng of recombinant mouse GM-CSF (Biolegend, San Diego, CA, USA) diluted in 20 μL PBS with 0.2% Bovine Serum Albumin (BSA Fraction V; GE Healthcare, Little Chalfont, United Kingdom), on the day of vaccination and the consecutive three days, in the same injection site as the vaccine. The control mice received a placebo consisting of 20 μL PBS with 0.2% BSA. Two different vaccination schemes were applied. Mice received either three shots of Infanrix® IPV within 9 weeks, and one shot of Boostrix Polio® 9 months thereafter (Fig. 1a). Alternatively, some mice received two and some received three shots of Infanrix® IPV within 9 weeks and were sacrificed 7 days after they had received their last shot (7 days after the second, or 7 days after the third shot; Fig. 1b), and splenocytes were isolated. With each shot, mice received either the GM-CSF or placebo treatment as described above. Blood of the studied mice was collected regularly from the *Superficial temporal* vein in BD Microtainer® SST™ tubes (BD Biosciences, San Jose, CA, USA) and serum was separated according to manufacturer’s instructions.

### 2.3 Diphtheria- and tetanus-specific antibody determination by ELISA

ELISA analysis was performed as previously described in detail [23]. Adjustments for the assessment of murine sera were made. In brief, microtiter plates were coated with 1 μg/mL diphtheria or tetanus toxoid (Statens Serum Institute, Copenhagen, Denmark) and blocked with 0.01 M Glycin. Serum samples were tested in duplicates. Peroxidase-labeled rabbit anti-mouse IgG antibody (Merck KGaA) was used as secondary antibody. IgG antibodies were quantified in arbitrary units (AU)/mL. A high titer serum pool from mice that had received three shots of Infanrix® IPV within 9 weeks, and were bled 14 days thereafter, was used as standard. The standard curve started at a 1:250 dilution and continued in a 1:2 serial dilution (in PBS/1% BSA) down to a final dilution of 1:16,000 (7 dilutions in total). The detection limits of the assays were 10.966 AU/mL for diphtheria-specific antibodies and 4.918 AU/mL for tetanus-specific antibodies, and values below this concentration were set to 5.483 and 2.459 AU/mL.

### 2.4 Isolation of cells

Spleen, lymph nodes, and muscle tissue were digested with Liberase™ Research Grade (Roche, Basel, Switzerland) DNAse I (Roche) for 30 min, smashed through a Falcon® 70 mm cell strainer (Corning, Corning, NY, USA) and washed with RPMI1640 with L-Glutamine and 25 mM Hepes (Lonza) supplemented with 10% fetal calf serum (FCS, Merck KGaA). Muscle tissues of the mice were scaled before cell isolation. Isolated cells from the spleen underwent erythrocyte lysis by incubating them for 5 min in 10 mL lysis buffer (155 mM NH_4_CL, 10 mM KHCO_3_, 0.1 mM EDTA pH 7.4; all Merck KGaA) at RT.

### 2.5 Flow cytometry

Cytokine production of diphtheria- or tetanus-specific CD4^+^ T cells was induced by re-stimulation of 2 million splenocytes with 10 μg/mL diphtheria or tetanus toxoid and 1 μg/mL anti-CD28 (clone: 37.51; BD Biosciences) at 37 °C for 6 h, with 10 μg/mL Brefeldin A (Merck KGaA) added after the first hour of stimulation. Live/dead staining was done with Zombie Violet™ Fixable Viability Dye (Biolegend). Surface staining was done with anti-CD3-BV510™ (clone: 17A2; Biolegend) and anti-CD4-PE/Cy7 (clone: RM4-4; Biolegend). Intracellular staining was done with anti-IFN-γ-PE (clone: XMG1.2; Biolegend), anti-TNF-α-FITC (MP6-XT22; Biolegend), anti-IL2-APC (clone: JES6-5H4; Biolegend), anti-IL4- FITC (clone: 11B11; Biolegend), anti-IL6-FITC (clone: MP5-20F3; ThermofischerScientific, Waltham, MA, USA), anti-IL10-APC (clone: JES5-16E3; Biolegend).

To study DCs, live/dead staining was done with Zombie Violet™ Fixable Viability Dye and isolated cells were stained subsequently with anti-CD3-PerCP-Cy™5.5 (clone: 145-2C11; BD Biosciences), anti-CD11b-APC-Cy™7 (clone: M1/70; BD Biosciences), anti- CD11c-BV510 (clone: HL3; BD Biosciences), anti-CD19-PerCP-Cy™5.5 (clone: 1D3; BD Biosciences), anti-CD40-PE-Vio770 (clone: FGK45.5; MiltenyiBiotec, Bergisch Gladbach, NW, Germany), anti- CD80-APC (clone: 16-10A1; BD Biosciences), anti-CD86-APC (clone: PO3.3; MiltenyiBiotec), anti-CD274-PE (clone: MIH5; BD Biosciences), anti-MHC-II-FITC (clone: M5/114.15.2; Thermofis-cherScientific), and anti-NKp46-PerCP-eFluor™710 (clone: 29A1.4; ThermofischerScientific).

All flow cytometry data were acquired using the FACSCanto™ II cytometer (BD Biosciences), exported as FCS3.0 files and analyzed using the FlowJo® software version 10.0.7 (FlowJo LLC, Ashland, OR, USA).

### 2.6 Determination of DCs in lymph node and muscle tissues

The total number of CD11b^+^ DCs was determined using CountBright™ absolute counting beads (cat.: C36950; Lot.: 1715265; ThermofischerScientific) as described in the user’s manual, 140 μL of cell suspension from lymph nodes and 190 μL of cell suspension from muscle tissue were stained with antibodies for flow cytometry. Immediately before the cells were acquired at the flow cytometer, 20 μL of counting beads were added. The CD11b^+^ DC concentration per mL and mg muscle was calculated according to this formula:

$$\begin{array}{l} \left(\left(\frac{\;numberof\;DCs}{\;numberofbeads\;}\times \frac{20,400\frac{\text{ beads }}{20}\mu \text{L}}{\;volumeofstainedcells\;\left(\mu \text{L}\right)}\right)\times 1000\right)\\ ÷\;weightofmuscletissue\;\left(\text{mg}\right)=\;numberof\;\frac{\frac{\text{DCs}}{\text{mL}}}{\text{mg}}\text{ muscle } \end{array}$$

The DC concentration per lymph node was calculated according to this formula:

$$\begin{array}{l} \left(\begin{array}{c} \;\frac{numberofDCs}{numberofbeads}\times \frac{20,400\frac{\text{ beads }}{20}\mu \text{L}}{volumeofstainedcells\;\left(\mu \text{L}\right)}\; \end{array}\right)\times 140\;\mu \text{L}\\ \text{ = }numberofDCsperlymphnode\; \end{array}$$

### 2.7 Statistical analysis

Group wise comparisons of diphtheria- and tetanus-specific antibodies were performed with two-way ANOVA for repeated measurements, using treatment and time as independent factors and antibodies as dependent variable. The main effect of treatment was reported.

A general linear model for repeated measurements was used to study the main effect of age on the production of diphtheria- and tetanus-specific antibodies following vaccination. Treatment and age were thereby used as ‘between-subjects’ and time as ‘within-subject’. Only the main effect of age was reported. Further subgroup analysis was done with two-way ANOVA for repeated measurements using treatment and time as independent factors and antibodies as dependent variable. Only the main effect of treatment was reported. Because of multiple comparisons, *p*-values were adjusted with Bonferroni-Holm correction.

Mann-Whitney U test was applied for all other statistical comparisons. The level of significance for all tests was considered α = 0.05.

IBM® SPSS® Statistics Ver.24.0.0.0 (IBM, Armonk, NY, USA) was used for the general linear model and GraphPad Prism® Ver.5.01 (GraphPad Software Inc., La Jolla, CA, USA) was used for all other statistical analyses and graphical representations.

## 3 Results

### 3.1 GM-CSF enhances diphtheria-specific antibody response following vaccination

The first objective was to test if *in vivo* application of GM-CSF influences the production of diphtheria- and tetanus-specific antibodies after vaccination. Young mice were vaccinated *i.m*. with commercially-available multivalent vaccines containing diphtheria and tetanus toxoid, according to the vaccination scheme shown in Fig. 1a. To model a primary vaccination series in children, they received three shots of Infanrix® IPV within 9 weeks, and one shot of Boostrix Polio® 9 months thereafter. The mice additionally received 100 ng GM-CSF together with each shot at the injection-site of the vaccine, as well as on the three consecutive days after each shot. A control group received a placebo consisting of PBS with 0.2% BSA (Fig. 1a). Antibodies with specificity against diphtheria and tetanus toxoids were assessed, and from here on are referred to as diphtheria-specific and tetanus-specific antibodies. Mice were bled regularly throughout the experiment and diphtheria- and tetanus-specific antibodies in the sera were determined by ELISA.

**Fig. 2:**
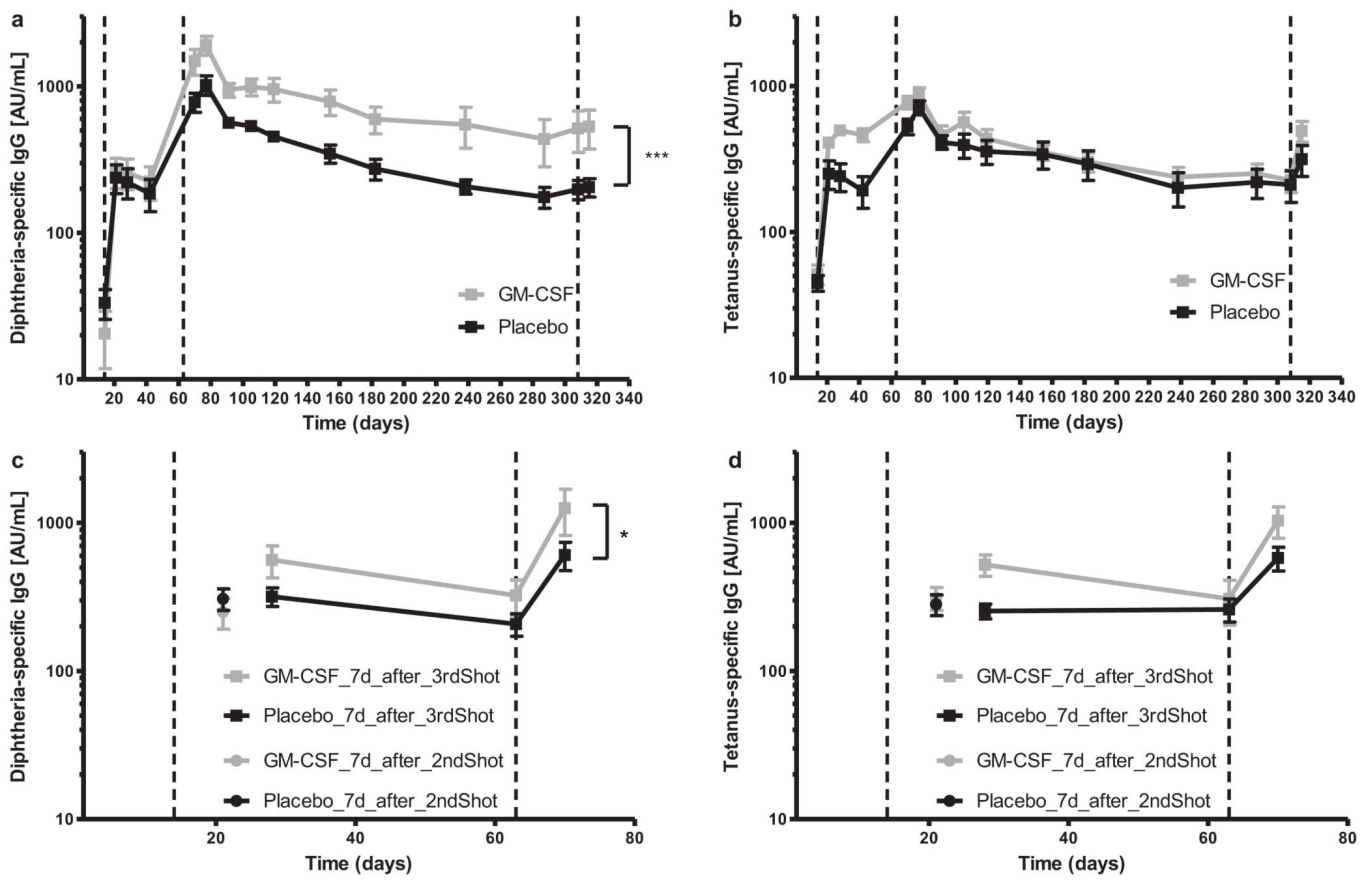
Diphtheria- and tetanus-specific antibodies following vaccination with GM-CSF. One group of mice were vaccinated with three shots of Infanrix® IPV (day1, day 14, day 63; dashed lines) and one shot of Boostrix Polio® (day 308; dashed lines) with simultaneous application of GM-CSF or placebo on the day of vaccination, and the consecutive three days (a and b). Another group of mice were vaccinated with either two or three shots of Infanrix® IPV (day1, day 14, day 63; dashed lines) and GM-CSF or placebo on the day of vaccination and the consecutive three days afterwards (c and d). Mice were bled regularly throughout the study, and (a and c) diphtheria- and (b and d) tetanus-specific IgG antibodies from blood serum were determined by ELISA and displayed in arbitrary units (AU). Two-way ANOVA for repeated measurements was performed with a sample size of n = 5–6 per treatment. The main effect of treatment is reported. *p < 0.05; ***p < 0.001.

After the first three vaccine shots, GM-CSF-treated mice had higher levels of diphtheria-specific antibodies when compared with the placebo group. Subsequent to reaching an antibody-plateau 14 days after completing the primary vaccination series, the placebo-group exhibited a faster decline of diphtheria-specific antibodies than the GM-CSF group (Fig. 2a). Both treatment groups displayed a slight increase in antibody production after receiving the final booster shot. Interestingly, although tetanus-specific antibody levels were different between the two treatment groups for the period between the second and the third shot of Infanrix® IPV, antibody concentrations were later similar (Fig. 2b).

In the next experiment we focused on the cellular immune response to vaccination, while simultaneously studying the production of diphtheria- and tetanus-specific antibodies. We followed a slightly different protocol, in which young mice were vaccinated *i.m*. with Infanrix® IPV with simultaneous application of GM-CSF or placebo, and were sacrificed either 7 days after the second, or 7 days after the third shot (Fig. 1b). Blood was taken regularly, and diphtheria- and tetanus-specific antibodies in sera were determined by ELISA. There was no difference in diphtheria- and tetanus-specific antibody production after the second shot (Fig. 2c and d). GM-CSF-treated mice once again had a stronger diphtheria-specific antibody production compared to the placebo group after receiving all three vaccine shots (Fig. 2c), whereas there was no difference in tetanus-specific antibodies (Fig. 2d). We conclude that simultaneous *in vivo* application of GM-CSF and multi-valent tetanus/diphtheria vaccines enhances diphtheria-, but not tetanus-specific antibody production.

### 3.2 CD4^+^ T cell response after GM-CSF treatment

We then analyzed whether *in vivo* application of GM-CSF together with vaccination leads to a stronger T cell response against diphtheria and tetanus. Young mice were vaccinated *i.m*. with either two or three shots of Infanrix® IPV with simultaneous application of GM-CSF or placebo. Splenocytes were isolated either 7 days after the second vaccine shot or 7 days after the third vaccine shot and re-stimulated *in vitro* with diphtheria and tetanus toxoids (Fig. 1b). The cytokine production of CD4^+^ T cells was studied by flow cytometry and cytokine-producing CD4^+^ T cells are referred to as diphtheria- or tetanus-specific CD4^+^ T cells.

**Fig. 3:**
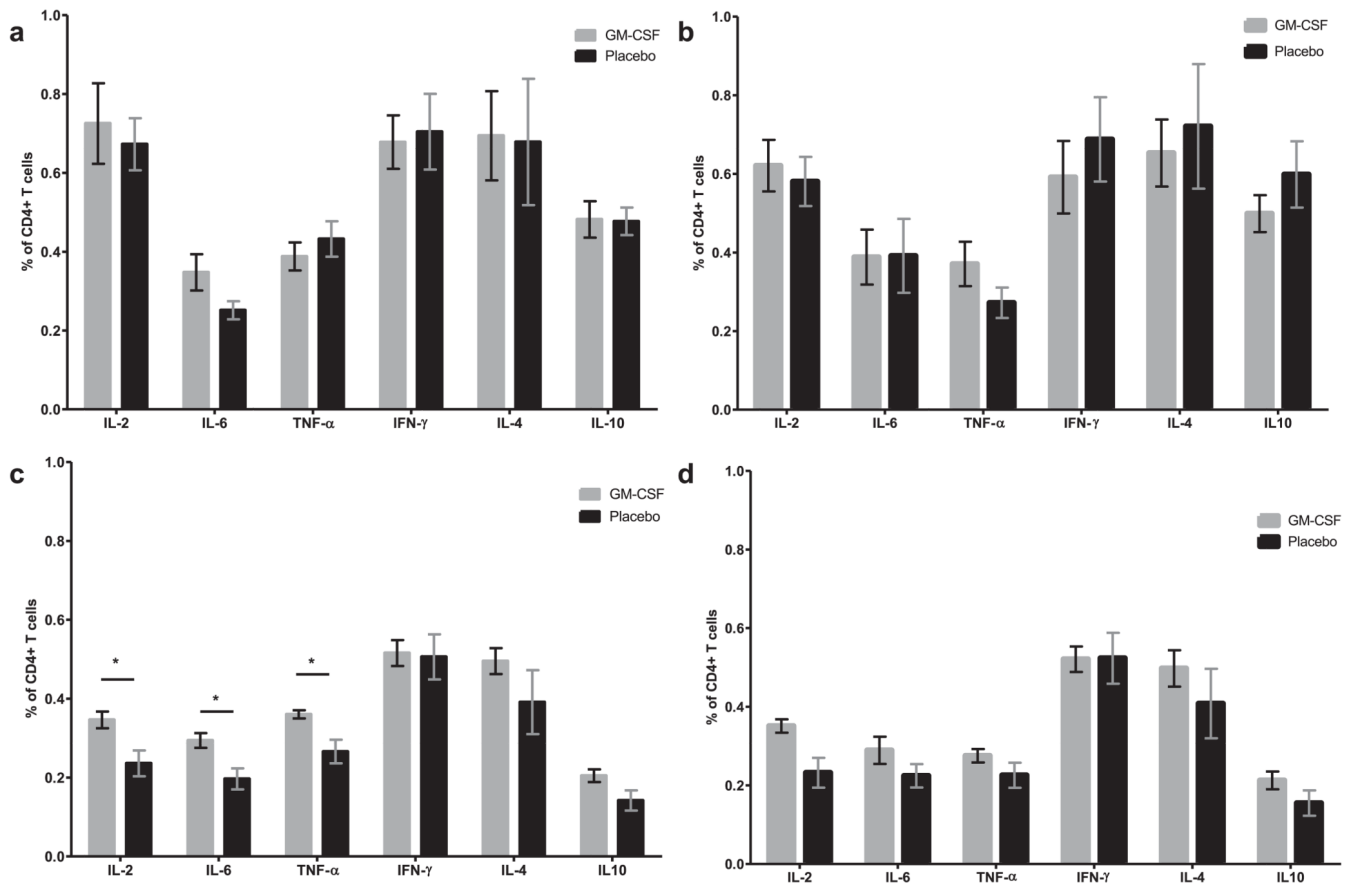
Diphtheria- and tetanus-specific CD4^+^ T cells following vaccination with GM-CSF. Mice received a primary immunization series with either two or three shots of Infanrix® IPV and GM-CSF or placebo as shown by the immunization scheme in Fig. 1b. Splenocytes were isolated either 7 days after the second shot (a and b), or seven days after the third shot (c and d), and re-stimulated with diphtheria (a and c) or tetanus (b and d) toxoid. Production of IL-2, IL-6, TNF-α, IFN-γ, IL-4, and IL-10 in CD4^+^ T cells was measured by flow cytometry. Mann-Whitney U test was performed with a sample size of n = 5–6. *p < 0.05.

No differences were detected between the percentages of cells producing the cytokines measured in the CD4^+^ T cells from GM-CSF- and placebo-treated mice following stimulation with either tetanus or diphtheria toxoid, 7 days after the second vaccine shot (Fig. 3a and b). Mice which had received all three shots of Infanrix® IPV and were sacrificed 7 days after the third shot had significantly more diphtheria-specific CD4^+^ T cells producing IL-2, IL-6, or TNF-α when treated with GM-CSF than placebo-treated controls (Fig. 3C). In contrast, there was no difference between the percentages of CD4^+^ T cells producing the cytokines in the two treatment groups when cells were stimulated with tetanus toxoid (Fig. 3d).

### 3.3 Influence of GM-CSF on dendritic cells

**Fig. 4:**
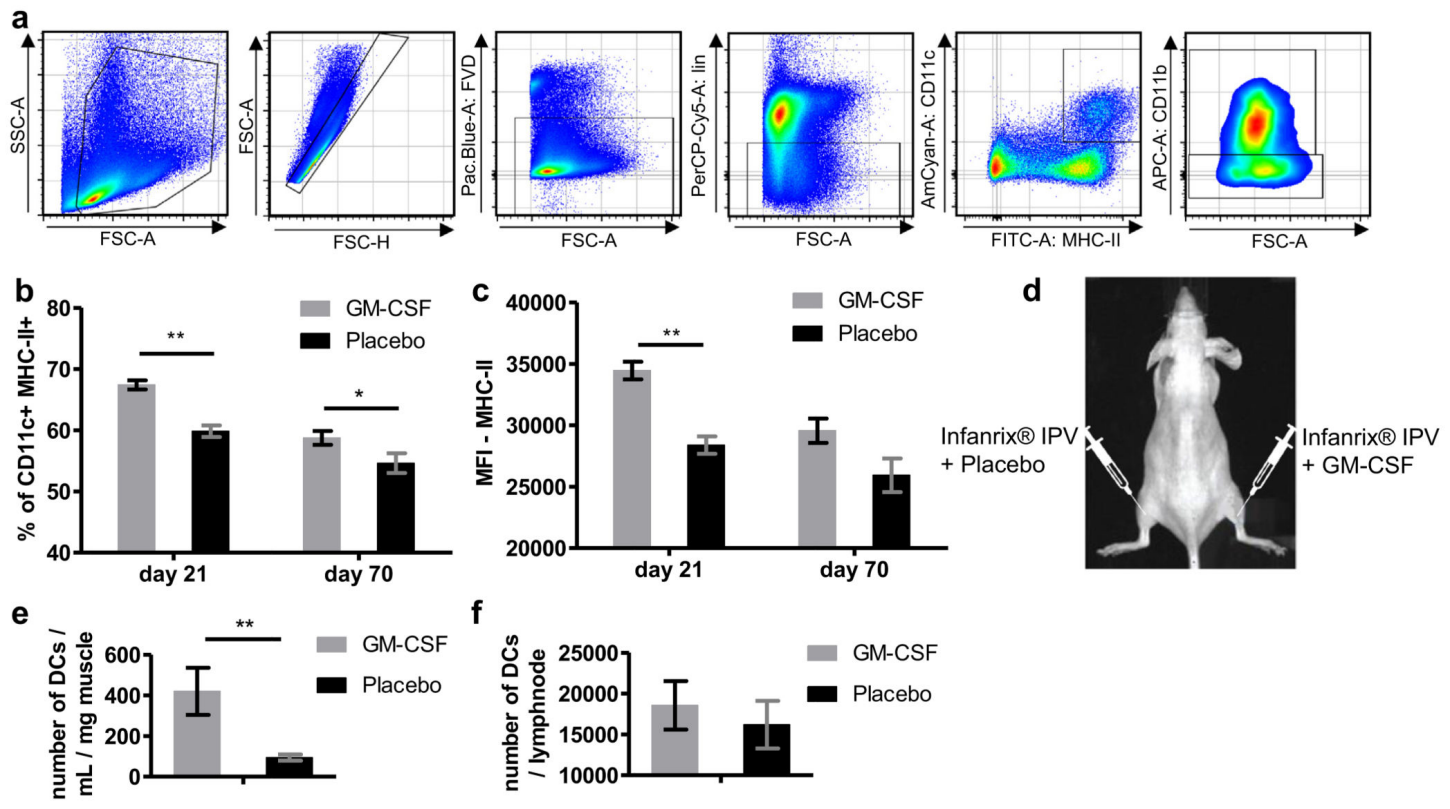
CD11b^+^ DCs after *in vivo* GM-CSF treatment. (a) Gating strategy for DCs. The fixable viability Dye Zombie Violet™ (FVD) was used as live/dead marker. NKp46, CD3, and CD19 were used as lineage markers (lin). DCs are referred to as CD11c^+^, MHC-II^+^ and CD11b^+^. (b and c) Mice received either two or three shots of Infanrix® IPV and GM-CSF or placebo as shown by the immunization scheme in Fig. 1b. and splenocytes were isolated 7 days after the last vaccine shot (either day 21 or day 70) and DCs were studied by flow cytometry. (b) Proportions of CD11b^+^ DCs from the total CD11c^+^ MHC-II^+^ population are shown. (c) Expression of MHC-II on CD11b^+^ DCs is shown in mean fluorescence intensity (MFI). (d-f) Mice received one shot of Infanrix® IPV *i.m*. in to the left and right legs *(M. biceps femoris)*. Additionally, they got injected with 100 ngof GM-CSF in to the right leg and a placebo in to the left leg (same injection site as the vaccine). Mice were sacrificed 24 h after receiving the injections, cells were isolated from each muscle tissue as well as from the popliteal lymph nodes and the number of DCs were studied by flow cytometry. (d) Graphic illustration of experimental setup is shown. Number of DCs in muscle tissue (e) and popliteal lymph node (f) were compared between the legs receiving GM-CSF and the ones with the placebo treatment. Mann-Whitney U test was performed with a sample size of n = 6. *p < 0.05; **p < 0.01; (For interpretation of the references to colour in this figure legend, the reader is referred to the web version of this article.)

As a next step, we studied the influence of *in vivo* GM-CSF application on splenic DCs subsequent to vaccination with Infanrix® IPV. Splenocytes from young mice were isolated either 7 days after the second or 7 days after the third vaccine shot, and studied by flow cytometry. Cells were gated as shown in Fig. 4a. CD11b^+^ DCs have a predominant role in the activation of CD4^+^ T cells and were thus analyzed [24].

GM-CSF-treated mice had higher proportions of CD11b^+^ DCs than placebo-treated mice after the second and third shot of the multivalent vaccine (Fig. 4b). There was also a difference in the expression of the MHC-II molecule on the surface of CD11b^+^ DCs between the two treatment groups. GM-CSF-treated mice had a stronger MHC-II expression after the second shot. After the third shot, MHC-II was slightly, but not significantly, higher in GM-CSF-treated mice (Fig. 4c).

We then studied DCs in greater depth, in particular whether the number of CD11b^+^ DCs at the injection site was influenced by GM-CSF. Mice received one shot of Infanrix® IPV *i.m*. into each leg, and additionally one shot of GM-CSF into the right leg and one shot of placebo into the left leg (same injection site as Infanrix® IPV, Fig. 4d). Mice were sacrificed 24 h after injection and cells were isolated from the muscles of both legs and from both popliteal lymph nodes. The total numbers of CD11b^+^ DCs were then determined by flow cytometry. The muscle tissue from the legs into which GM-CSF had been injected contained significantly more CD11b^+^ DCs per mg muscle than the control legs, which had been injected with the placebo (Fig. 4e). The numbers of CD11b^+^ DCs in the popliteal lymph nodes of both legs were not significantly different (Fig. 4f).

### 3.4 GM-CSF improves diphtheria-specific antibody response in old mice

Finally, we studied whether *in vivo* application of GM-CSF can improve the production of diphtheria- and tetanus-specific anti-bodies following vaccination in aged mice. Seventeen month and 8 week old mice were vaccinated *i.m*. with three shots of the mul-tivalent Infanrix® IPV, according to the vaccination scheme shown for the primary immunization in Fig. 1a. Mice additionally received GM-CSF or placebo *i.m*. in the same spot (Fig. 1a). Mice were bled regularly throughout the experiment until 120 days after the first shot. Diphtheria- and tetanus-specific antibodies in the sera were determined by ELISA.

**Fig. 5:**
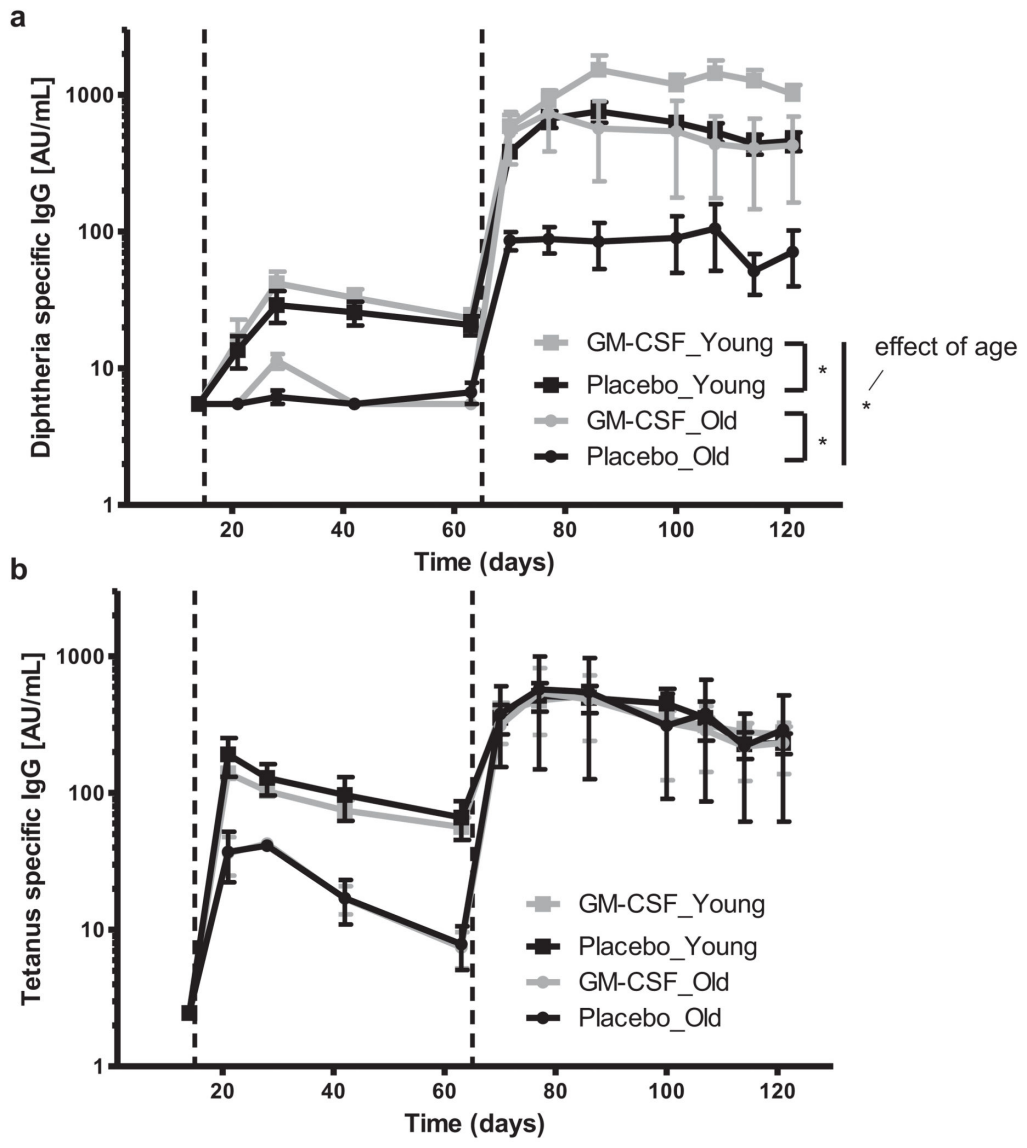
Diphtheria- and tetanus-specific antibodies in young and old mice following vaccination with Infanrix® IPV and GM-CSF. Mice were vaccinated with three shots of Infanrix® IPV (day1, day 14, day 63; dashed lines) with simultaneous application of GM-CSF or placebo on the day of vaccination and the consecutive three days. They were bled regularly throughout the study and (a) diphtheria- and (b) tetanus-specific IgG antibodies from blood serum were determined by ELISA and displayed in arbitrary units (AU). The main effect of age on the production of diphtheria- and tetanus-specific antibodies was studied by a general linear model for repeated measurements. Two-way ANOVA for repeated measurements was used for subgroup analysis to study the main effect of treatment on antibody production. *p*-values were adjusted for multiple comparisons with Bonferroni-Holm correction. The sample size was n = 4–6 per treatment. *p < 0.05.

GM-CSF- and placebo-treated young mice had similar diphtheria-specific antibody titers until the third vaccine shot. Thereafter the GM-CSF group had significantly more diphtheria- specific antibodies compared to the placebo group (Fig. 5a). Exactly the same response pattern was found in aged mice, which initially had lower diphtheria-specific antibody levels than young mice. In general, aging had an effect on the production of diphtheria-specific antibodies. However, with the application of GM-CSF, it was possible to increase the production of diphtheria-specific antibodies in old mice to the same level as observed in young placebo-treated mice (*p* = 0.764). Diphtheria-specific antibody concentrations in old GM-CSF treated mice were still lower than that of young GM-CSF treated mice. Again, GM-CSF application had no influence on the production of tetanus-specific antibodies, as there was no difference in tetanus-specific antibody levels between the placebo- and the GM-CSF-treated group in both, young and aged mice (Fig. 5b). Interestingly there was no effect of age on the production of tetanus-specific antibodies.

## 4 Discussion

Multivalent vaccines have the advantage of a reduced number of injections and doctor’s visits for immunizations. In the case of multivalent tetanus/diphtheria vaccines, it has been demonstrated that adults are more often unprotected against diphtheria than against tetanus [3–5]. This situation is more pronounced in the elderly population, for which the consequences of diphtheria infection would be disastrous. Diphtheria is still a dangerous infectious disease for all age groups, as shown by a recent outbreak in Yemen with more than 333 cases and a mortality of more than 10% [25]. Interestingly 39% of the suspected cases had been vaccinated against diphtheria, indicating that there is a need to improve the current vaccination strategy and/or the vaccine. To define a potentially successful strategy we tried to link several immunological parameters with antibody concentrations against diphtheria in young and elderly adults [5]. In doing this, we found a positive correlation between GM-CSF production by CD4^+^ T cells and diphtheria-, but not tetanus-specific antibodies. We were thus wondering whether *in vivo* administration of GM-CSF would affect the immune responsiveness to the diphtheria-component in multi-valent vaccines. We now showed for the first time that *in vivo* application of recombinant GM-CSF boosted the cellular as well as the humoral immune response to diphtheria, while the response to tetanus was unaltered. Remarkably, we also succeeded in improving the relatively low immune response to diphtheria in GM-CSF treated-aged mice to the same level as the one of placebo treated-young mice. However, there was still a difference between young and old GM-CSF treated mice.

Mice had increased diphtheria-specific antibody concentrations when GM-CSF was applied in addition to a multivalent tetanus/ diphtheria vaccine. GM-CSF has not yet been combined with diphtheria vaccine or multivalent vaccine, but with monovalent vaccines where GM-CSF was either incorporated into the vaccine formulation or applied together with the vaccine. GM-CSF induced increased antibody titers to H5N1 influenza virus, *Chlamydia trachomatis*, and HIV [16,26,22]. GM-CSF only had a stimulatory effect on immunization against *C. trachomatis* when applied at the same site as the antigen [26], indicating that GM-CSF is only advantageous for the immune response in close vicinity to the vaccine. We also studied the humoral response to the tetanus-component of the multivalent vaccine and did not observe any effect of GM-CSF on the production of tetanus-specific antibodies. Therefore it seems that GM-CSF does not boost the immune responsiveness of all components of the vaccine. We did not analyze antibodies against pertussis or polio in this study, as both types of antibodies were difficult to assess and interpret in a previous publication [3]. In this context it is also of interest that different adjuvants had different effects on the production of tetanus- and diphtheria-specific antibodies in mice [27]. This also suggests that the two antigens respond differently to co-stimuli and that GM-CSF plays an adjuvant role for diphtheria, but not for tetanus toxoid. Biophysical comparisons additionally demonstrated that the tetanus toxoid is three times bigger than the diphtheria toxoid [28]. Due to this fact, more epitopes may be presented to T cells by APCs, thereby also promoting better antibody production compared to the smaller diphtheria antigen, which likely has fewer epitopes, and may be more susceptible to co-stimulation. However, the exact mechanism by which GM-CSF boosts the diphtheria-, but not the tetanus-specific immune response, is a question that needs to be addressed in future studies.

GM-CSF also stimulated the diphtheria-specific CD4^+^ T cell response. Mice vaccinated with GM-CSF had increased proportions of diphtheria-specific CD4^+^ T cells producing IL-2, IL-6, and TNF-α compared to placebo-treated mice, whereas again, there was no difference in the proportion of tetanus-specific CD4^+^ T cells. IL-6 is beneficial for the generation of T follicular helper cells and thus indirectly supports the production of high affinity antibodies by plasma cells [29–31]. Furthermore, IL-6 is known to influence T-helper cell differentiation into Th2 cells by an auto-feedback loop with upregulation of IL-4 production [32]. Both IL-2 and TNF-α would also strengthen the immune response to the vaccine [33]. Our data support previous publications which demonstrate that GM-CSF boosts antigen-specific CD4^+^ T cell responses [18,20].

GM-CSF is also a good stimulator of DCs [34,35]. Hence, we were interested in the effects of GM-CSF on DCs in our vaccinated mice. We found higher proportions of CD11b^+^ DCs in the spleen of GM-CSF-treated mice, which also had a higher expression of MHC-II. Upregulation of MHC-II expression is known to occur with DC maturation, and is essential for antigen presentation to CD4^+^ T cells [36]. The recruitment of CD11b^+^ DCs to the injected muscle immediately after vaccination was also increased by GM-CSF treatment, indicating that GM-CSF exerted a strong chemotactic effect on DCs when co-applied with diphtheria toxoid. This is in accordance with previous reports which emphasized the importance of GM-CSF as a stimulator of antigenic response by supporting the recruitment and migration of leucocytes, and in particular DCs, to the local administration site [15,19].

The fact that GM-CSF is improving the diphtheria-, but not the tetanus-component of the multivalent vaccine is of interest, as there is no urgent need to boost protection against tetanus either in young or in elderly adults [3–5]. However, there is a strong need to improve protection against diphtheria, especially in the elderly population, and our results demonstrate for the first time, that GM-CSF can mediate an enhanced diphtheria-specific immune response in an aged setting. Targeting DCs may therefore be a promising approach to design vaccines that specifically meet the needs of the aging immune system. The vaccination scheme applied in aged mice with three shots of Infanrix® IPV is not meant to be used in adult humans. The reason for using this protocol was to directly compare the effect of GM-CSF on the immune response to vaccination in the two age groups. Irrespective of the promising results, using cytokines as adjuvants is presently expensive and does therefore not represent a very likely vaccination strategy. Biotechnological as well as logistical improvements may lower the costs, which could make this approach a realistic alternative to conventional vaccines.

In summary, our study demonstrates that local administration of recombinant GM-CSF can improve the immune response to the diphtheria-component in a multivalent tetanus and diphtheria toxoid containing vaccine, while the response to the tetanus-component remains unaltered. The exact mechanism is still unclear, but it is most likely DCs that are primarily affected. Our results are also promising in terms of future vaccine development customized for the elderly, in which only single components of multivalent vaccines may need to be improved.
